# Viscoelastic Rheological Behaviors of Polypropylene and LMPP Blends

**DOI:** 10.3390/polym13203485

**Published:** 2021-10-11

**Authors:** Feichao Zhu, Sohail Yasin, Munir Hussain

**Affiliations:** 1 Department of Textile Engineering, College of Textile Science and Engineering, Zhejiang Sci-Tech University, Hangzhou 310027, China; zhufeichao@zstu.edu.cn; 2 Zhejiang Provincial Key Laboratory of Industrial Textile Materials and Manufacturing Technology, Zhejiang Sci-Tech University, Hangzhou 310027, China; 3 MOE Key Laboratory of Macromolecular Synthesis and Functionalization, Department of Polymer Science and Engineering, Zhejiang University, Hangzhou 310027, China; munir88@zju.edu.cn

**Keywords:** LMPP, polypropylene, LAOS, rheology, Lissajous

## Abstract

Dynamic oscillatory shear testing is used to investigate polymeric viscoelastic behaviors. Small and large amplitude oscillatory shear tests are the canonical method for characterizing the linear and nonlinear viscoelastic behaviors of any polymeric material. With prominent and abundant work on linear viscoelastic studies, the nonlinear behavior is evasive in terms of generating infinite higher harmonics in the nonlinear regime. For this reason, intrinsic nonlinearities from large amplitude oscillatory shear (LAOS) studies have recently been used for insights on microstructural behaviors. This study is carried out for linear and nonlinear viscoelastic behavior with a main focus on LAOS of isostatic polypropylene (iPP) and relatively new low molecular weight and low modulus polypropylene-based polyolefin (LMPP) blends. The morphological results showed reduced spherulitic crystal nucleus size and increased distribution in blends with increasing LMPP. The blends showed subtle linear viscoelastic responses with strong nonlinear mechanical responses to variant strain and stress compared to pure iPP. The intracycle strain thickening and intracycle strain stiffening of high-content LMPP blends were comparatively dominant at medium strain amplitudes.

## 1. Introduction

Isostatic polypropylene (iPP), classified as an important polymer, is often restricted due to relatively low toughness and high thermal expansion coefficients [1]. Blending and polymeric modifications are required to improve the properties. Blending of iPP with a low molecular weight and low modulus (LMPP) in small amounts [2,3,4], has been reported to produce finer and softer fiber products, such as nonwovens. LMPP is a relatively new polypropylene-based polyolefin developed by Idemitsu Petrochemical, Ltd., posing variant properties associated with conventional polypropylene. High crystallinity and melting point, which is attained using metallocene catalyst, reinforces the stereoregularity of the polymer [5]. Polyolefin elastomers provide elasticity, cohesive strength and adequate melt flow rate, yet show immiscibility, even when having similar monomer chemistry [6]. However, the properties only develop slowly after the application, as the high crystallinity is critical to bond stability over time [7]. Moreover, iPP and elastomer blends are heterogeneous at low temperatures, thus the properties are dependent on shape and size distribution of polyolefin spherulites of the blends [8]. In terms of improved iPP products, morphology and viscoelastic properties of the blends are essentially important when blended with newer polymers. Moreover, determination of the rheological properties is considered crucial for the fundamental understanding of blends processability [9,10].

iPP, being thermoplastic polymer, requires high processing temperatures to reach appropriate melt viscosity. To analyze viscosity, a capillary rheometer is considered suitable for measuring rheological properties of iPP blends at high temperatures and with wide shear rate ranges. It provides easy to handle high viscous melts at high temperatures and is industrially applicable by being similar to actual processing extrusion or injection molding with the same shear rates and flow geometry [11,12]. Immiscibility due to low entropy of mixing, phase separation and low interaction with each other are known hurdles while blending. Indeed, the properties of the blends are dependent on physical properties, including the morphology and the chemistry of the polymers, showing their interactions. Degree of polymeric dispersion/distribution and tendency of phase separation can be distinguished rheologically. This work provides effects of LMPP in iPP blends morphology accompanied by viscoelastic characterization. Viscoelastic characterization with dynamic rheological analysis of polymeric materials is important [13,14,15] for prior final designing and processing of any product [16,17]. In viscoelastic characterization, application of a large amplitude oscillatory strain (LAOS) marks non-sinusoidal stress responses, acknowledged as nonlinear response. Later, stress decompositions by Fourier transform [18] on time domain stress response, resulting power spectrum, are frequently applied for the interpretation of LAOS data [19,20,21]. LAOS measurements have shown higher sensitivity to instantaneous structural changes in polymeric solutions and melts [1,14]. Qualitative data from LAOS is graphically presented with Lissajous plots, where stress and strain are shown as a function of elastic and viscous responses. Next, the intercycle and intracycle responses focusing on elastic and viscous nonlinearities are investigated at different strain amplitudes.

## 2. Materials and Methods

The isotactic polypropylene (iPP), s2040 (Ziegler–Natta) with a molecular weight (MW) 2.0 × 10^5^ g/mol was provided by SECCO Petrochemical Ltd., Shanghai, China. The LMPP (s901) was provided by Idemitsu Kosan Co, Ltd. Tokyo, Japan, with MW 1.3 × 10^5^ g/mol. The characterization data for the material used are presented in Table 1. The polymeric materials used in this work were of laboratory grade samples. Melt flow rate index (MFR) was 50 g/10 min for LMPP. The mixing ratios of iPP/LMPP by (wt/wt): 100/0, 95/5, 90/10, 85/15, 80/20, 75/25, were prepared and defined as 5%, 10%, 15%, 20% and 25% LMPP blends. The morphological effects of iPP/LMPP blends were investigated by comparing 100% iPP and 100% LMPP.

Mechanical blends of iPP/LMPP at the compositions of interest were prepared with the help of a mechanical blender by Giant Co., Ltd. Nanjing, China and mixed for 2 min at room temperature. Melt-blended samples of the homopolymers with various compositions were prepared by a twin-screw extruder (TSE-30A Ruiya extrusion system Co., Ltd., Nanjing, China) with an L/D = 40, and the processing speed of extruder was 60 rpm at 210 °C.

Polarized optical and light microscope (POM/PLM) was used to characterize the growth of iPP/LMPP spherulites and crystals morphology. For that, a small piece of sample was cut and compressed manually in a compression machine at ~30 bars. The samples were placed on a hot stage and preheated at 25 °C to 200 °C at a rate of 50 °C/min for 2 min until the samples were melted. Reaching 200 °C, the samples were cooled at 120 °C at a rate of 50 °C/min for 15 min isothermal temperature. At a temperature of 120 °C, POM images were captured. A Leica DM2700P transmission/reflection polarizing microscope (Beijing, China) was used. Hot-pressed blends into tablets (~0.2 mm) were observed at room temperature after cooling and crystallization.

The capillary rheology of the blends was carried out at three varying temperatures: 190, 200 and 210 °C. The blends were added to the barrel after two preloads (0.3 MPa) and two heat-rheological tests (total 5 min) later. The shear rate range was 100–6000 s^−1^, and capillary diameter 1 mm with length to diameter ratio L/D = 16. A die of zero shear stress was used. Dynamic linear rheological responses of the blends at 150 °C were measured on a strain-controlled rheometer (ARES-G2, TA Instruments, New Castle, DE, USA) with a plate geometry of 25 mm in diameter. Angular frequency (ω) sweep tests, from ω = 100 rad/s to 0.01 rad/s at γ = 0.5%, were conducted. Similarly for LAOS, oscillatory data were obtained in parallel plate geometry of 25 mm in diameter at 150 °C at frequency (ω) 1.0 rad s^−1^ from the same rheometer. Rheological response characterization of pure LMPP was restricted for its low melting temperature compared to iPP, thus it was not included in the analysis. The strain amplitudes (γ_0_) were set from 0.1% to 1000%. In the linear regime, when a sinusoidal strain γ(t) = γ_0_ sin(ωt) is applied at a given γ_0_ to the blends, a sinusoidal stress σ(t) = σ_0_ sin(ωt + δ) with stress amplitude σ_0_ and phase angle δ is generated [14,22,23,24]. Decomposition of σ(t) into σ(t) = σ’ sin(ωt) + iσ” cos(ωt) yields the first-order harmonics, i.e., elastic (G’ = σ’/γ_0_) and viscous moduli (G” = σ”/γ_0_), which are used for linear viscoelasticity characterization. Here σ’ and σ” are in- and out-of-phase stress responses, respectively. Whereas, in nonlinear regimes, the higher order harmonics depending on γ_0_ and ω [25] become more essential [26]. For decomposing stress into n^th^-order harmonics G’_n_(γ_0_,ω) and G’’_n_(γ_0_,ω), Fourier series [19], σ(t;ω,γ_0_) = γ_0_Σ_n,odd_ [G’_n_(ω,γ_0_) sin(nωt) + iG”_n_(ω,γ_0_) cos(nωt)], is used. Lissajous plots for σ’ against γ and σ” against $\dot{\gamma }$ and Fourier transform spectrum are evaluated to quantify the nonlinearity of given iPP/LMPP blends [27].

## 3. Results

**Figure 1:**
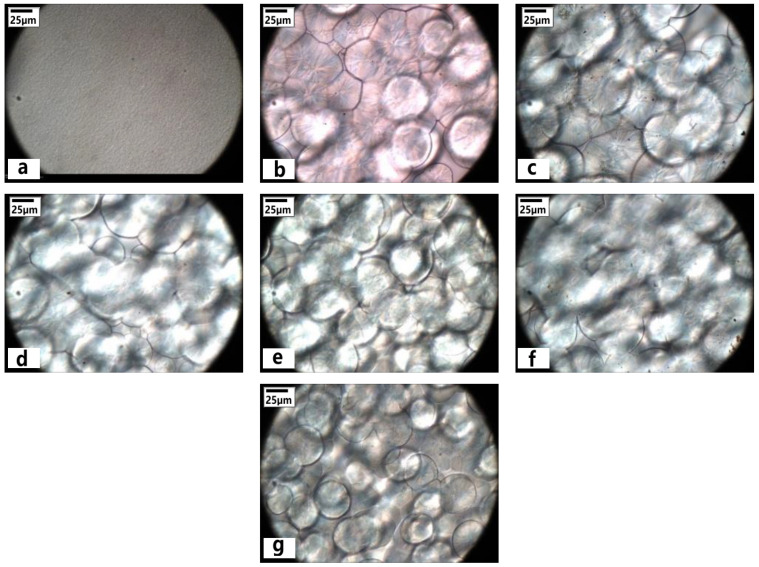
Morphology of spherulites for pure (**a**) LMPP, (**b**) iPP, (**c**) 5%, (**d**) 10%, (**e**) 15%, (**f**) 20% and (**g**) 25% LMPP blends.

**Figure 2:**
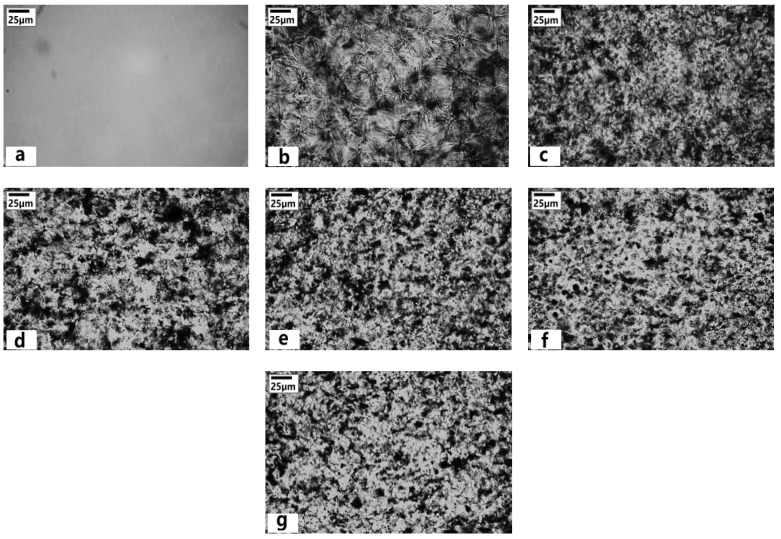
Morphology of pure (**a**) LMPP, (**b**) iPP, (**c**) 5%, (**d**) 10%, (**e**) 15%, (**f**) 20% and (**g**) 25% LMPP blend crystals (adapted from [29]).

POM was used to investigate the growth of the spherulite in iPP/LMPP blends. The spherulites of the blends were characterized at 120 °C for 15 min isothermally. From Figure 1, influence of LMPP on the size, structure and types of spherulite crystals is visible, while pure LMPP appeared with no spherulites, indicating the crystallinity being very low [3,28]. On the other hand, the pure iPP contains many spherulites that show higher crystallinity than LMPP. However, spherulites with increased LMPP content to iPP sharpen the pellets and round the shape with clear boundaries with bright light birefringence within the spherulites. The crystalline morphology of iPP/LMPP blends can be seen in the polarized light microscope image in Figure 2. It was found that the spherulites of pure iPP were obvious with large size (~50 μm) and high crystallinity, while being low for pure LMPP with no obvious spherulites. Addition of LMPP to iPP reduces the crystal nucleus size affecting iPP spherulites in high dispersion/distribution. The molecular chain in LMPP enters into the iPP macromolecular chain, which alters the iPP crystallization process [2,3,4]. During the crystallization of pure iPP, the spherulites grow and the compression inhibits the increase of the diameter. The addition of LMPP may have a dilution effect and may act as a plasticizer, increasing space between spherulites of the blends till the content increased to 15%. Reaching the threshold, the size of spherulites appeared to be decreasing in 25% LMPP blend (Figure 2g). Similarly, we found the degree of crystallinity of blends increases for 5–15% LMPP contents and then progressively decreases for 20–25% with possible diluent LMPP molecular suppression entanglement between the iPP molecules [3]. The mobility restriction of iPP molecules during the crystallization has also been reported for the decrease in crystallinity of iPP blends [3,29].

**Figure 3:**
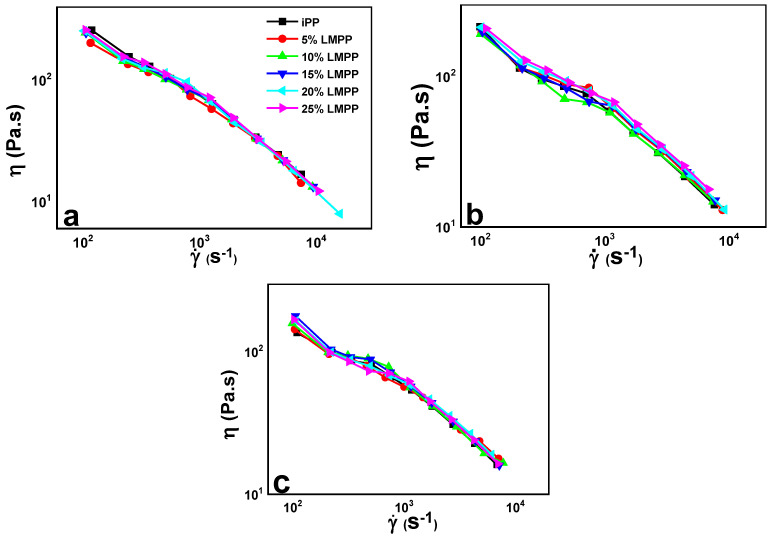
Capillary rheology plot of shear viscosity (η) against shear rate ($\dot{\gamma }$) for pure iPP, LMPP and blends at (**a**) 190, (**b**) 200 and (**c**) 210 °C.

Rheological evaluation differentiates interaction effects on polymer chains from phase separation, cross-linking network structure in the melt and incompatibility, providing information on the morphology evolution and reconfiguration of the network structure [30]. Capillary rheological behavior of the iPP and blends with different LMPP composition are shown in Figure 3. At shear rates near zero, the apparent viscosity of the melts slightly rose with increasing LMPP contents. At 5% LMPP content, the viscosity is the lowest (Figure 3a); at 25% the apparent viscosity is near to pure iPP 250 Pa.s. At lower shear rates, the viscosity of blends appears to be reducing down to 150 Pa.s.; this later improves slightly. Between 1000–3000 s^−1^, the decline is eased, reaching to 25 Pa.s. Similar behavior was seen in blends at higher temperatures, 200 and 210 °C. The increasing temperatures and decreasing shear viscosity may be incurred because of enhanced macromolecule chain movement, also decreasing the apparent viscosity.

**Figure 4:**
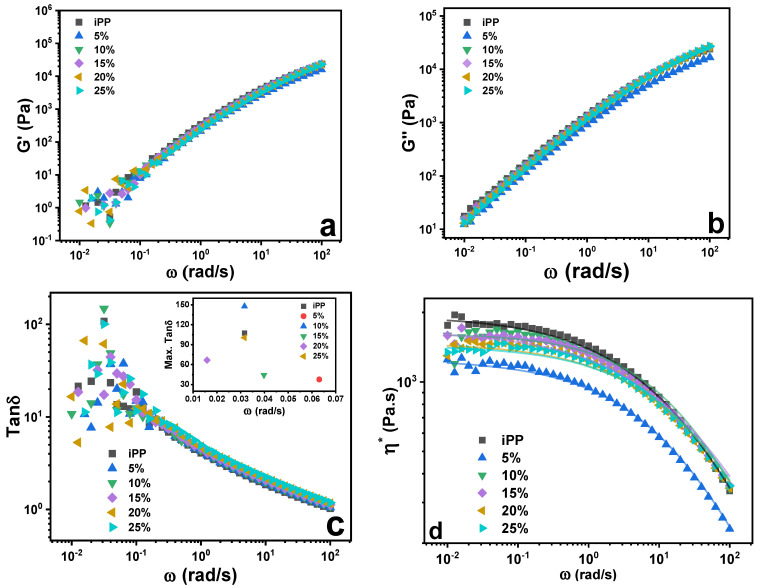
Dynamic rheological response for storage modulus (G′) (**a**), loss modulus (G″) (**b**), loss tangent (tanδ) with inset (max. tanδ) (**c**) and complex viscosity (*η), line ‘fitted with Carreau-Yasuda model (**d**) as a function of angular frequency (ω) at γ = 0.5% and 150 °C for pure iPP, LMPP and blends.

Similarly, in linear rheology of blends, storage modulus (G′) and loss modulus (G″) as a function of frequency (ω) of iPP/LMPP blends showed slight changes compared to pure iPP; such behavior is known in cross-linked polymers [31,32]. The moduli G″ > G′ were dominant across the majority of ω range, which indicates the predominant viscous response of the blends and the pure iPP. Overall improved ω dependence of blends was seen more in G″ than G′, while the loss tangent (tanδ) showed arbitrary behavior at lower ω (Figure 4c). The max. tanδ was higher for pure iPP and the blends with 15% and 25% LMPP contents at lower ω.

The complex viscosity (*η) of the pure iPP and LMPP blends is shown in Figure 4d; the data are fitted with the Carreau-Yasuda model [33], suggesting pure iPP, and all blends demonstrated shear-thinning behavior. Addition of lower M_w_ polymer LMPP lowers the *η of the blends compared to pure iPP, which may be ascribed to the better polymeric adhesion between them.

**Figure 5:**
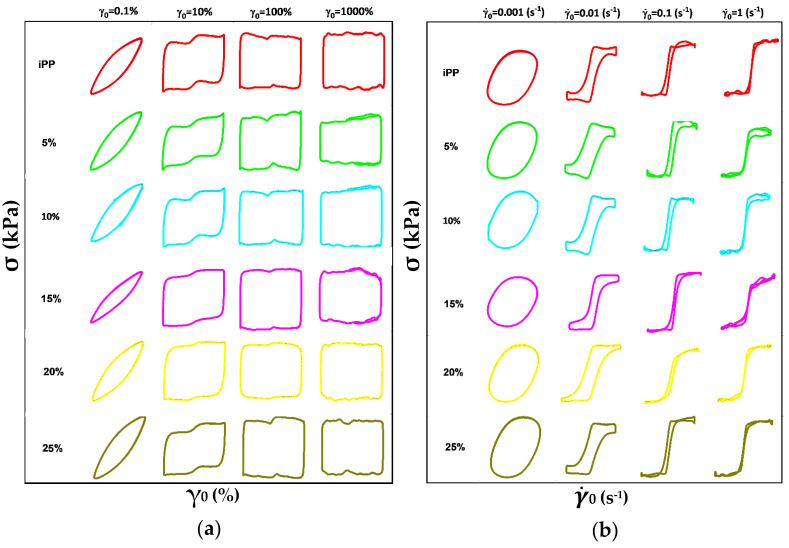
Lissajous curve plots of dynamic stress (σ) against (**a**) dynamic strain (γ_0_) and (**b**) strain-rate amplitude ($\dot{\gamma }$_0_) for pure iPP, LMPP and blends at 1.0 rad s^−1^.

G′ and G″ being linear viscoelastic moduli are not sufficiently enough to determine the material’s responses, as the stress wave is no longer sinusoidal beyond the linear regime [34]. For that purpose, LAOS studies are carried out. Figure 5a shows the elastic Lissajous curves of the σ against γ_0_; at lower γ_0_ the blends kept constant shape, with no response with the addition of LMPP. Whereas at higher γ_0_ the blends kept the elliptical shape which widened reaching the rectangular shape, referring to the plastic characteristic of the materials [14,22]. Whereas from Figure 5b, at each chosen $\dot{\gamma }$_0_ the shape of the Lissajous plot distorts as $\dot{\gamma }$_0_ increases. It can be seen from Figure 5 that blends with 20% LMPP have the sharpest and clearest shape and boundaries at all γ_0_ and $\dot{\gamma }$_0_, ascribed to better physical polymeric interactions.

**Figure 6:**
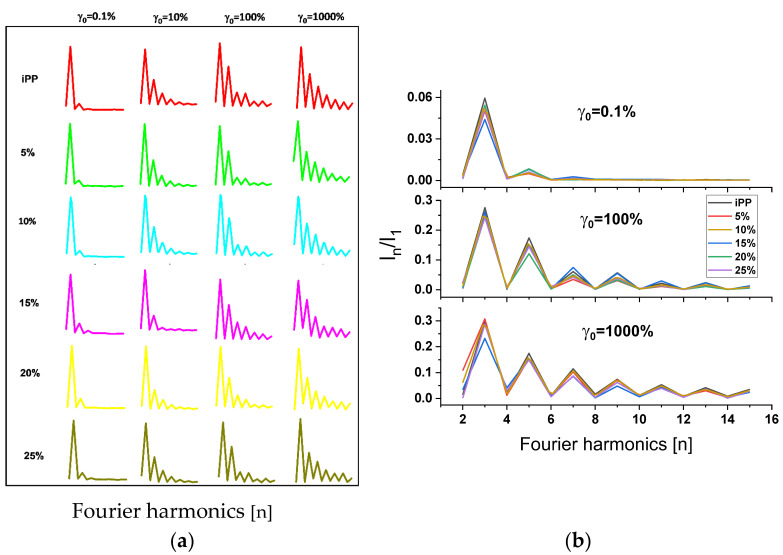
Fourier spectra at different dynamic strains (γ_0_) for pure iPP, LMPP and blends at 1.0 rad s^−1^ (**a**), (**b**) showing comparative intensities of blends at given γ_0_.

In Figure 6, Fourier transform spectrum shows odd high-order harmonics for nonlinearity from dominant responses of the first harmonic in all samples. From the relative intensities (I_n_/I_1_), the values of I_3_/I_1_, I_5_/I_1_ and I_7_/I_1_ in the blends reduce at lower γ_0_, compared to pure iPP. Blends with 15, 20 and 25% LMPP contents showed slightly higher intensities than pure iPP (Figure 6b), indicating LMPP affects viscoelasticity nonlinearities.

**Figure 7:**
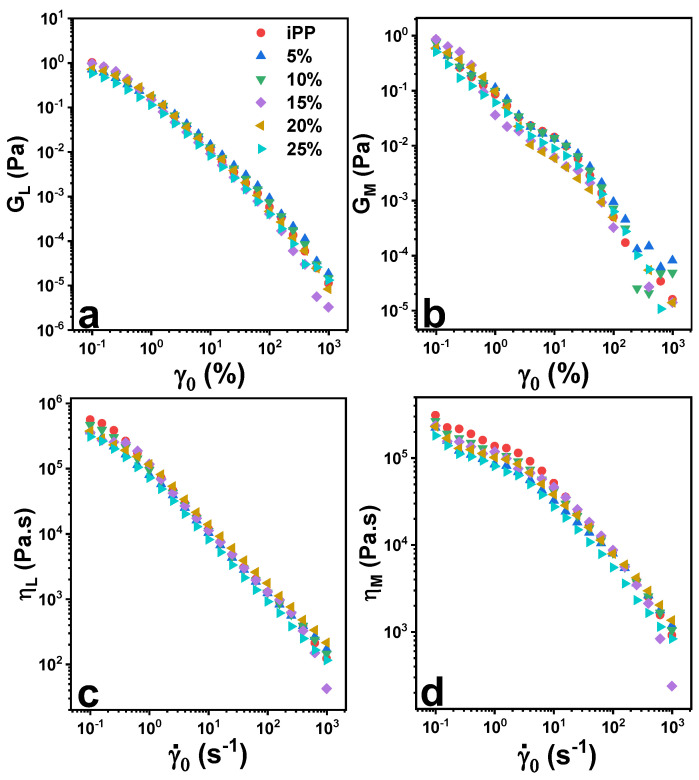
Large-strain elastic modulus (G_L_; (**a**)), minimum-strain elastic modulus (G_M_; (**b**)), large strain-rate dynamic viscosity (η_L_; (**c**)) and minimum strain-rate dynamic viscosity (η_M_; (**d**)) as a function of strain-rate amplitude $\dot{\gamma }$_0_ and strain amplitude γ_0_ under LAOS stress for pure iPP, LMPP and blends at 1.0 rad s^−1^.

Under LAOS stresses, the blends demonstrated subtle large-strain elastic modulus (G_L_) and large strain-rate dynamic viscosity (η_L_), while minimum-strain elastic modulus (G_M_) and minimum strain-rate dynamic viscosity (η_M_) were influenced with the addition of LMPP as shown in Figure 7. Dependence of G_L_ and η_L_ with increasing γ_0_ and $\dot{\gamma }$_0_ in linear to nonlinearity regime was obvious, while G_M_ and η_M_ were dominant in linear regime. With both G_L_ and G_M_ decreasing with increasing γ_0_, the blends and pure iPP exhibit overall intercycle strain softening. Whereas decreasing η_L_ and η_M_ showed intercycle shear thinning behavior in blends and pure iPP.

**Figure 8:**
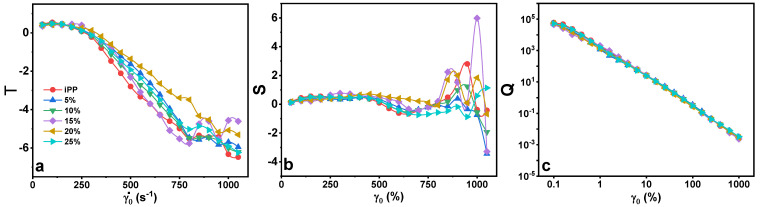
Intracycle strain thickening (T; (**a**)), intracycle strain stiffening (S; (**b**)) and Q parameter (**c**) as a function of strain-rate amplitude $\dot{\gamma }$_0_ and strain amplitude γ_0_ under LAOS stress for pure iPP, LMPP and blends at 1.0 rad s^−1^.

In Figure 8, the intracycle and intercycle nonlinear responses were quantified as strain-stiffening ratio (S = 1 − GM/GL) and shear thickening ratio (T = 1 − ηM/ηL) [14,35], where blends showed arbitrary behavior at higher γ_0_, similarly with subtle Q parameter. It is a ratio of the intensity of the 3rd harmonic response of elastic stress to the 1st (I_3/1_), divided by the strain amplitude squared $\gamma_{0}^{2}$, calculated as Q(ω,γ_0_) ≡ I_3/1_/$\gamma_{0}^{2}$ [36], providing information on polymer systems with two material interfaces [37]. Such intrinsic nonlinear parameters have been used to assess dispersion/distribution, polymeric interface interactions in blends and quantification of blends mixing [36,37,38].

## 4. Discussion

The spherulite shape and size are the obvious characteristics [39]. Spherulitic growth rate is related to the crystallization temperature and is nonlinear in non-isothermal conditions of the polymeric blends. The POM shown in Figure 1, the spherulitic growth rate of the blends presents regular growth with the addition of LMPP contents, affecting the size and shape of the spherulites. With the addition of LMPP, there is a change in the crystallization temperature of the blends, affecting the melt viscosity, crystal diffusion rate of the chains and their segmental mobility (Figure 2) [40]. LMPP is a low tacticity polypropylene, known for great spinability and elongation [41]. In our previous study, the blends with 5%, 10% and 15% LMPP contents showed crystallinity at 43.2%, 43.9% and 42.5%, while the numbers were 40.2% and 16.5% for pure iPP and LMPP. Similarly, the mid content ratios showed enhanced mechanical properties, while showing the highest elongation for 25% LMPP blend [3]. Overview of crystallinity and thermal properties of the blends is given in Table 2, calculated previously [3] from differential scanning calorimeter (DSC) and X-ray diffraction (XRD). From DSC analysis, the crystallization temperature (tC) of blends decreases with increased LMPP content, while the tM and melt enthalpy (△H_f_) of pure LMPP is lower, indicating low crystallinity and different crystal type from iPP [3,7]. Whereas the average spherulite size of blends appeared enlarged with the LMPP addition at lower contents (~61 μm), this later reduces at higher contents significantly (~52 μm).

It is demonstrated that the shear viscosity is stable at given temperatures, with increased LMPP content, keeping the rheological flow (capillary rheology) preserved similar to iPP, as shown in Figure 3a–c. This suggests that addition of LMPP in iPP blends will not affect processing extrusion or injection molding. With improved chain mobility the iPP/LMPP melts undergo low shear viscosity at all given temperatures without any flow resistance. It can be deduced that pure iPP and its blends with LMPP demonstrate shear-thinning behavior.

In the linear rheology, the ω dependent linear G′ and G″ in Figure 4, with initial dominant G′ > G″ and slight G′ < G″ at higher ω in all the blends, structural relaxation was demonstrated. Figure 5 describes the viscoelastic perspective of the materials, which is difficult to distinguish from the G′ and G″ only. From the qualitative information from LAOS rheological behavior, the blends showed enhanced nonlinearities with the addition of LMPP. The ascending addition of LMPP to the blends does not significantly influence the shape compared to pure iPP, indicating the stabilized structure. However, the blends at variant γ_0_ and $\dot{\gamma }$_0_ showed sharper edged Lissajous figures, with slightly smoother final shapes. Such changes are associated with the enhanced dissipation of the mechanical energy within a cycle [42]. From the Fourier transform rheology in Figure 6, the higher-order odd harmonics for relative intensities (I_n_/I_1_) are normalized by the fundamental one; here however, the affected viscoelasticity nonlinearity of the blends may relate to but not be determined by the amount of LMPP.

For the nonlinear viscoelastic moduli shown in Figure 7, all samples may demonstrate shear-thickening trend; however, blend responses were sensitive when it comes to η_M_ and G_M_, yielding a LAOS fingerprint. The LAOS fingerprinting is used to define specific viscoelastic nonlinearities [27]. The blends showed primarily elastic nonlinearities, however; affected η_M_ at lower $\dot{\gamma }$_0_, samples showed predominantly strain-rate softening behavior.

From Figure 8, the elastic nonlinearity perspective of the blends in initial to medium γ_0_ showed weak intracycle softening (S < 0), while showing intracycle hardening (S > 0) at higher γ_0_ range, where 20 and 25% predominate compared to pure iPP. The blends’ behavior towards viscous nonlinearity was less arbitrary, where all blends and pure iPP featured shear thinning (T < 0) at medium to high $\dot{\gamma }$_0_. This showed dependence of the intracycle viscoelastic behavior with regards to dimensionless but prominent S nonlinearity in high-content blends compared to T, where there was a gradual decline.

## 5. Conclusions

In accordance with the previous study, degree of crystallinity of blends increases for 5–15% LMPP contents and then progressively decreases for 20–25%. Similarly, the addition of LMPP in iPP blends affects the spherulitic growth rate in size and shape with clear boundaries and bright light birefringence. This spherulitic effect was apparent in high-content LMPP blends, which also reduces the crystal nucleus size and promotes molecular dispersion/distribution in iPP. In terms of responses from capillary rheology, shear-thinning behavior was observed in pure iPP and all blends. The dynamic linear rheological responses were subtle in all specimens, whereas variant intrinsic viscoelastic nonlinearities were observed from LAOS tests. High-content LMPP blends posed intracycle strain-thickening and intracycle strain-stiffening behavior at medium strain amplitudes. From this LAOS study, change in the viscoelastic nonlinearities of iPP blended with LMPP was observed in both, strain and strain-rate space.

## Figures and Tables

**Table 1:** Ingredients used for the blends.

Materials	Type	Catalyst	^1^ MW (g/mol)	^2^ MW/MN	Density (kg/m^3^)	MFR (g/10 min)	^3^ TM (°C)
iPP	Isotactic	Ziegler Natta	200,000	3.5	900	36	165.5
LMPP	Atactic	Metallocene	130,000	2	870	50	79.1

^1^ MW: molecular weight; ^2^ MW/MN: molecular weight/molecular number; ^3^ TM: melting temperature.

**Table 2:** Crystallinity and thermal properties of the blends. Data from [3].

iPP/ LMPP	^1^ TC (°C)	TM (°C)	^2^ △H_f_ (J/g)	Crystallinity (%)	Spherulite Size (μm)
100/0	116.3	159.1	91.0	40.2	50
0/100	40.1	79.1	20.6	16.5	0
95/5	116.0	158.2	84.2	43.2	58
90/10	115.8	159.1	77.0	40.9	61
85/15	115.3	157.4	72.1	42.5	60
80/20	114.2	156.5	68.2	38.8	60
75/25	114.0	155.3	65.0	38.3	52

^1^ TC: Crystallization temperature; ^2^ △H_f_: Melt enthalpy.

## Data Availability

The data presented in this study are available upon request from the corresponding author.
